# Phytochelatin synthase is required for tolerating metal toxicity in a basidiomycete yeast and is a conserved factor involved in metal homeostasis in fungi

**DOI:** 10.1186/s40694-015-0013-3

**Published:** 2015-03-28

**Authors:** Alaina M Shine, Viplendra PS Shakya, Alexander Idnurm

**Affiliations:** 1 Division of Cell Biology and Biophysics, School of Biological Sciences, University of Missouri-Kansas City, Kansas City, MO 64110 USA; 2 School of BioSciences, University of Melbourne, Melbourne, Vic 3010 Australia

**Keywords:** EC 2.3.2.15, Glutathione gamma-glutamylcysteinyltransferase, Heavy metal, Red yeast, Sporidiobolales

## Abstract

**Background:**

Phytochelatin synthase (PCS) is an enzyme that catalyzes the biosynthesis of phytochelatin from glutathione. Phytochelatins protect cells against the toxic effects of non-essential heavy metals, such as cadmium, and hence growth is restricted in the presence of these metals in mutants in PCS-encoding genes. PCS genes from fungi have been characterized in only two species in the Ascomycota, and these genes are considered sparsely distributed in the fungal kingdom.

**Results:**

A gene encoding a putative PCS was identified in *Sporobolomyces* sp. strain IAM 13481, a fungus that is a member of the Pucciniomycotina subphylum of the Basidiomycota. The function of this *PCS1* gene was assessed by heterologous expression in the Ascomycota yeasts *Saccharomyces cerevisiae* and *Schizosaccharomyces pombe*, and by mutating the gene in *Sporobolomyces*. The gene is required for tolerance to toxic concentrations of non-essential cadmium as well as the essential metal copper. Pcs1 homologs in fungi and other eukaryotes have putative targeting sequences for mitochondrial localization: the *S. pombe* homolog was fused to green fluorescent protein and it co-localized with a mitochondrial dye. Evaluation of the presence or absence of PCS and PCS-like homologs in the genome sequences of fungi indicates that they have a wide distribution, and the absence in most Ascomycota and Basidiomycota (the Dikarya) species can be explained by a small number of gene losses.

**Conclusions:**

The ecology of the species within the fungi carrying putative PCS genes, the phenotypes of phytochelatin synthase mutants in two major fungal lineages, and the presence of homologs in many non-Dikarya lineages parallel what is seen in the plant and animal kingdoms. That is, PCS is a protein present early during the evolution of the fungi and whose role is not solely dedicated to combating toxic concentrations of non-essential metals.

**Electronic supplementary material:**

The online version of this article (doi:10.1186/s40694-015-0013-3) contains supplementary material, which is available to authorized users.

## Background

Metal ions are cofactors that are required for many enzymes and transcriptional regulators, yet these same essential metals can become toxic at high concentrations, and non-essential metals are often toxic [1]. Organisms have different mechanisms to tolerate or combat exposure to high levels of metals. Understanding those mechanisms and then employing this information has many potential benefits. Amongst them, the knowledge can aid in bioremediation processes of environmental sites contaminated with metals, in modifying organism properties, or in using metals as antimicrobial agents.

One approach used by organisms to avoid metal toxicity is to chelate metal ions with another molecule to sequester the metal within the cell. Phytochelatins are small peptides that bind heavy metals. A gene encoding a phytochelatin synthase was identified in the late 1990s in three independent studies. In one, a wheat cDNA was cloned that conferred resistance to cadmium when expressed in budding yeast *Saccharomyces cerevisiae* [2]. Deletion of the homologous gene that was identified in the fission yeast *Schizosaccharomyces pombe* rendered the mutant sensitive to cadmium. In a second study, the phytochelatin synthase was identified by map-based cloning in *Arabidopsis thaliana* [3]. A deletion strain for the gene was also made in *S. pombe*, resulting in a strain that exhibited sensitivity to arsenic and cadmium. In the third study, the *A. thaliana* gene was identified by its expression in an *S. cerevisiae yap1*Δ strain through its ability to confer resistance to cadmium [4].

With a clear role in protecting against the harmful effects of metals, the original research and much subsequent research focused on the roles phytochelatin synthase have in non-essential metal toxicity, including the potential manipulation of the genes for bioremediation purposes. However, dealing with non-essential metals is unlikely to be the sole role of the phytochelatin synthases that have been identified, because they are found in organisms that are not exposed to those metal ions at high concentrations, a puzzle that has been noted previously [5,6]. More recently, a role in the homeostasis of essential metal ions has been defined because one phenotype of loss of PCS activity is zinc sensitivity in *A. thaliana* and in *S. pombe* in a Zn-homeostasis mutant background [7].

The fungi share many of the same habitats with plants, and, like plants, as individual organisms they have restricted capabilities to move location in response to environmental conditions. As addressed above, the analysis of the genome sequence of *S. pombe* revealed a homolog of phytochelatin synthase. Other homologs were not subsequently characterized in the fungi until recently when a *PCS1* homolog was identified in the genome sequence of the truffle-forming species *Tuber melanosporum* and noted in two other species of *Schizosaccharomyces* [8]. The fungi are a group of more than a million species [9,10]. The sparse distribution of PCS reported in the fungi led to the hypothesis that the *Schizosaccharomyces* PCS genes may have been acquired in those species by a horizontal gene transfer event [5].

This current research identified a putative phytochelatin synthase gene in a basidiomycete and aimed to test if the gene encoded a functional phytochelatin synthase. Analysis of genome sequences of fungi reveals a presence of PCS in many other fungal lineages outside of the Ascomycota and Basidiomycota, indicating a far wider distribution of this gene in the fungi than previously appreciated.

## Results

### *Sporobolomyces* sp. encodes a putative phytochelatin synthase homolog that can protect *S. cerevisiae* from toxic levels of heavy metals

A putative phytochelatin synthase (PCS) homolog was identified during the analysis and annotation of the genome sequence of a strain of *Sporobolomyces*. The rarity of this gene in reports from fungi triggered an investigation into the function of this gene in *Sporobolomyces*, and then the evolution of PCS homologs in the fungi.

**Figure 1:**
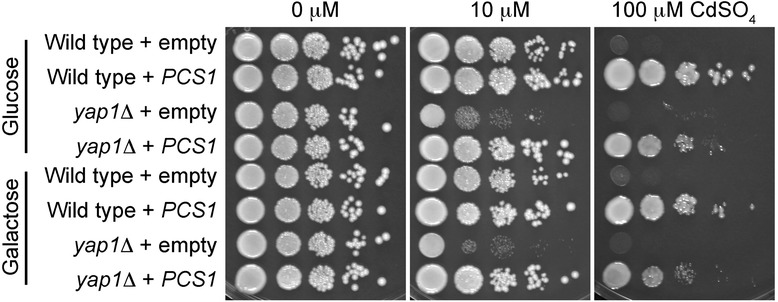
**Heterologous expression of** ***Sporobolomyces PCS1*** **in** ***S. cerevisiae*** **increases its resistance to cadmium.** The *PCS1* cDNA was expressed in a vector allowing induction in response to galactose. Wild type and the *yap1* mutant strain were transformed with this plasmid or the empty control and cultured overnight in YNB with either glucose or galactose as the carbon source. Ten-fold serial dilutions were plated onto YPD medium with or without cadmium sulfate and cultured 2 d at 30°C.

One approach used to clone phytochelatin synthases from plants was to express plant cDNAs in a *S. cerevisiae* strain with a *yap1* mutation and identify cadmium-resistant transformants. Yap1 is a transcription factor that controls the expression of genes required for protection against oxidative and other stresses, such as cadmium [11,12]. Expression of the PCS-encoding genes increased the resistance of this mutant to toxic metals. In an equivalent experiment, the *Sporobolomyces PCS1* cDNA was cloned into an *S. cerevisiae* plasmid that enables transcript induction in response to galactose in the medium. Wild type and *yap1* mutant strains containing this plasmid and the control empty plasmid were cultured overnight in media containing glucose or galactose, and plated onto medium supplemented with cadmium. The presence of the *Sporobolomyces* gene increased the resistance of both wild type and *yap1* mutant strains to cadmium (Figure 1). This also occurred under the non-inducing conditions in which there is residual expression from the *GAL1* promoter [13], indicating that even a small amount of PCS is able to combat cadmium toxicity. These findings are consistent with a role of the *Sporobolomyces* gene in biosynthesis of phytochelatin.

### The *Sporobolomyces pcs1* mutant is sensitive to high metal concentrations

**Figure 2:**
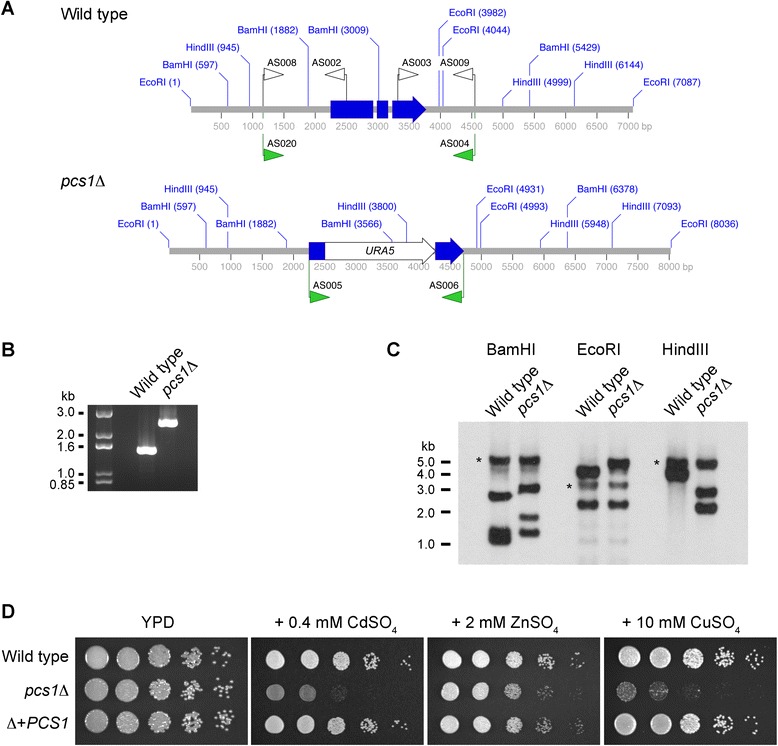
**Mutation of** ***PCS1*** **in** ***Sporobolomyces*** **causes sensitivity to metal stresses. (A)** Diagram of the DNA organization around the wild type and mutant allele of *PCS1*. The three exons of *PCS1* are the large blue boxes in the wild type, with the region replaced by the *URA5* marker illustrated with the white box. The positions of primers (AS###) are indicated by arrows. AS008-AS002 and AS003-AS009 were used to generate 5′ and 3′ fragments for targeted homologous replacement. Primers AS020-AS004 were used to amplify the probe used in the Southern blot, and primers AS005-AS006 used for the PCR in panel B. Restriction enzyme sites relevant to the Southern blot are provided along with the relative positions in parentheses. **(B)** PCR analysis using primers AS005-AS006 on the wild type and *pcs1* deletion strains. **(C)** Southern blot analysis of gene replacement of *PCS1* with the *URA5* gene. Genomic DNA was digested with BamHI, EcoRI or HindIII restriction enzymes and resolved on a 1% agarose gel. ^32^P-labelled DNA amplified with primers AS020-AS004 was hybridized to the blot. Note that the amplification of the probe yielded a second non-specific product, which hybridizes to the bands marked with *. **(D)** Phenotypes of the gene replacement strain compared to wild type and complemented (Δ + *PCS1*) strain. Ten-fold serial dilutions were plated onto YPD medium with or without supplements, and cultured for 3 d at 22°C.

Heterologous expression of the *PCS1* homolog of *Sporobolomyces* supported the hypothesis that this gene can synthesize protective molecules in fungi. In order to test the function of the gene directly in *Sporobolomyces*, a gene replacement strain was constructed (Figure 2A). Regions flanking the *PCS1* gene were amplified by PCR and used to generate an overlap product that was transformed into a *Sporobolomyces* uracil auxotroph. The mutant strain was identified by PCR analysis (Figure 2B), and gene replacement confirmed by Southern blot analysis (Figure 2C). As a control, a complemented strain was generated to include with phenotypic comparisons. For this purpose, the *URA5* gene was “recycled” for use as a transformation marker, by selection on 5-FOA medium for strains with spontaneous mutations. A wild type copy of *PCS1* was fused adjacent to the wild type copy of the *URA5* gene by overlap PCR, and this construct transformed into the *pcs1*Δ *ura5* strain to produce the complemented control strain.

Different metals were tested for their effects on the wild type, *pcs1* deletion mutant and complemented strains. Greatest sensitivity in the deletion mutant was found for cadmium and copper (Figure 2D). A slight decrease in growth in high levels of zinc was also observed. In contrast, no major difference was observed for toxic levels of cobalt chloride, sodium arsenite, iron sulfate, manganese chloride, or reactive oxygen species H_2_O_2_ and *t*-butyl-hydroperoxide.

The transcript levels of the *PCS1* gene in *Sporobolomyces* were examined by northern blot for the wild type strain cultured overnight in YPD alone or in the presence of three metals: copper, cadmium and zinc. No difference in transcript level was noted between any of these treatments or with the strain grown in YPD medium (data not shown). The regulation of phytochelatin synthase activity levels varies between species, although in many the transcript and protein are expressed constitutively with an induction of enzyme activity by direct interaction with the metal [14,15], and presumably a similar mechanism occurs in *Sporobolomyces*.

### *Sporobolomyces* sp. *PCS1* complements the phenotypes associated with loss of phytochelatin synthase from the ascomycete *S. pombe*

**Figure 3:**
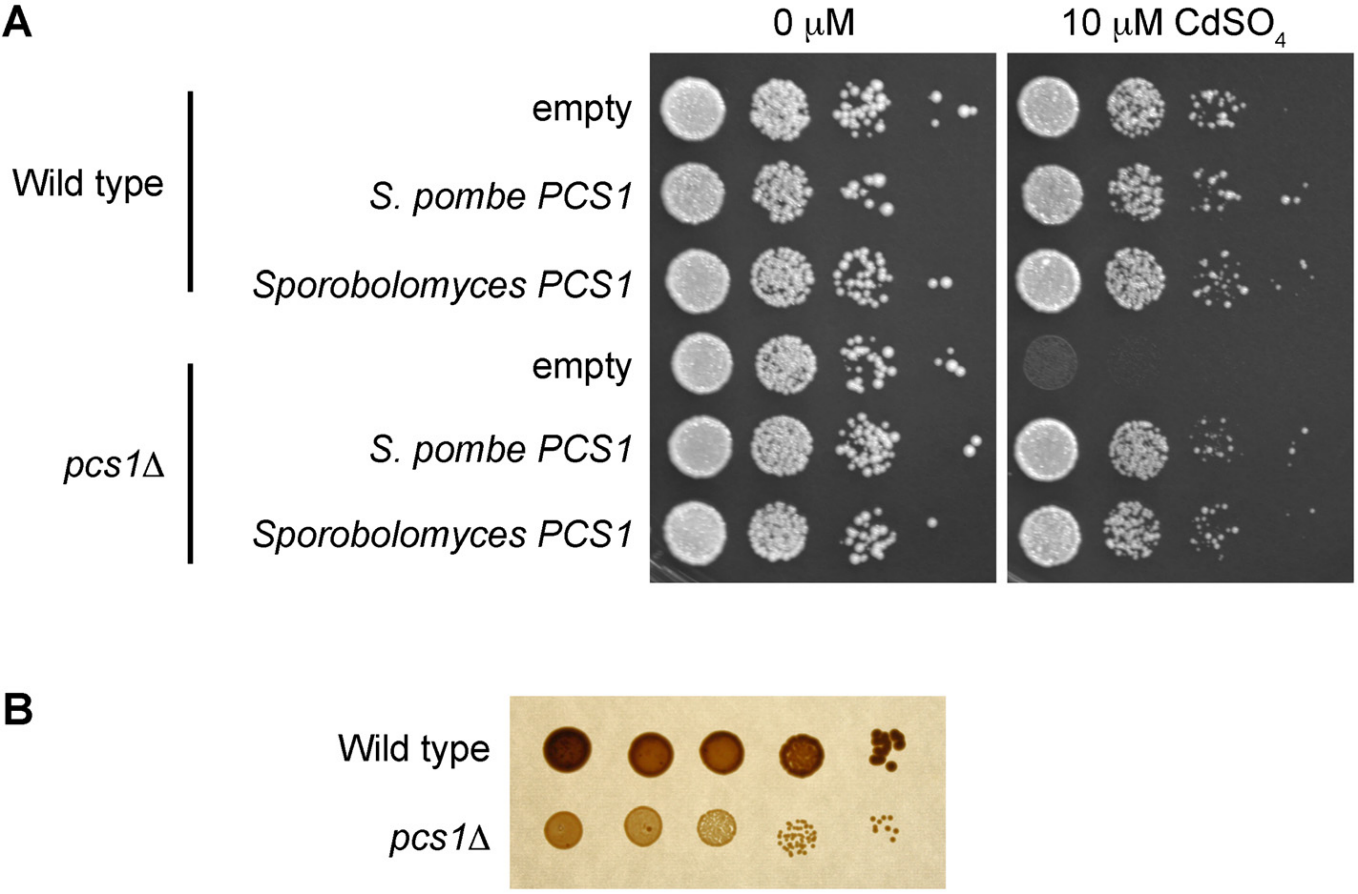
**The** ***Sporobolomyces PCS1*** **gene complements the** ***S. pombe pcs1*** **Δ phenotypes. (A)** Ten fold serial dilutions of cultures of *S. pombe* strains on YPD with or without 10 μM CdSO_4_, and cultured 3 d at 30°C. The wild type or *pcs1* deletion mutant strains contain the empty pREP42 plasmid or this plasmid including the cDNA clones of the *PCS1* homologs from *S. pombe* or *Sporobolomyces*. **(B)** The *pcs1* mutant has slower growth on YES + uracil (20 mg/L) + CuSO_4_ (0.625 mM) and an alteration in pigmentation. Growth of strains MM72-4A and AS8 after 6 d at 22°C.

To investigate the functions of *PCS1* more widely in the fungi and if the *Sporobolomyces* gene could complement the loss of PCS function, *S. pombe* was used. First, a gene mutant (*pcs1*Δ) was constructed in *S. pombe* that replaced the *pcs1* gene with the *KanMX* construct. The cDNA of *Sporobolomyces* and the ORF of *S. pombe* were amplified and cloned into a plasmid that enables a low level of expression of these genes when thiamine is present in media as used here [16]. The plasmids were transformed into the *S. pombe* deletion mutant, and growth tested on media containing cadmium (Figure 3A). The *S. pombe pcs1* mutant was sensitive to cadmium, as reported previously. This sensitivity was complemented by addition of the wild type copy of the gene from either *S. pombe* or *Sporobolomyces* into the mutant strain.

Testing growth under other metal conditions revealed that the *S. pombe pcs1* mutant also has phenotypes in the presence of copper. That is, there was increased sensitivity to growth on copper. In addition, at lower concentrations the wild type strain became pigmented, while the *pcs1* mutant strain had less pigmentation, indicating a change in copper homeostasis (Figure 3B).

### Phytochelatin synthase is localized in the mitochondria in *S. pombe*

Where PCS functions within the cell is largely unexplored, but one prediction is that it can be localized to the mitochondria [17,18]. The amino acid sequences of the *Sporobolomyces* and *S. pombe* homologs were examined with two software programs (Psort II and MitoProt) that predict subcellular localization, and both predicted mitochondrial localizations because of the presence of a putative mitochondrial targeting sequence at the N-terminus of the proteins.

To test its localization, fusions were created to produce proteins with Pcs1 at the N-terminus and GFP at the C-terminus, using both the *Sporobolomyces* and *S. pombe* homologs. The *Sporobolomyces* Pcs1-GFP fusion construct used the native promoter, and was transformed into the *pcs1*Δ strain. While the construct could complement the cadmium-sensitivity of the mutation, no fluorescence was observed for these strains (data not shown). To date, achieving a GFP signal in *Sporobolomyces* has been elusive, even with the use of a modified version of GFP that contains a *Sporobolomyces* intron that is functional in another basidiomycete yeast (i.e. *Cryptococcus neoformans*). Others working to express foreign genes in basidiomycetes have found that this may require optimizing constructs to alter the GC content of their DNA or include introns (see as examples [19-22]).

**Figure 4:**
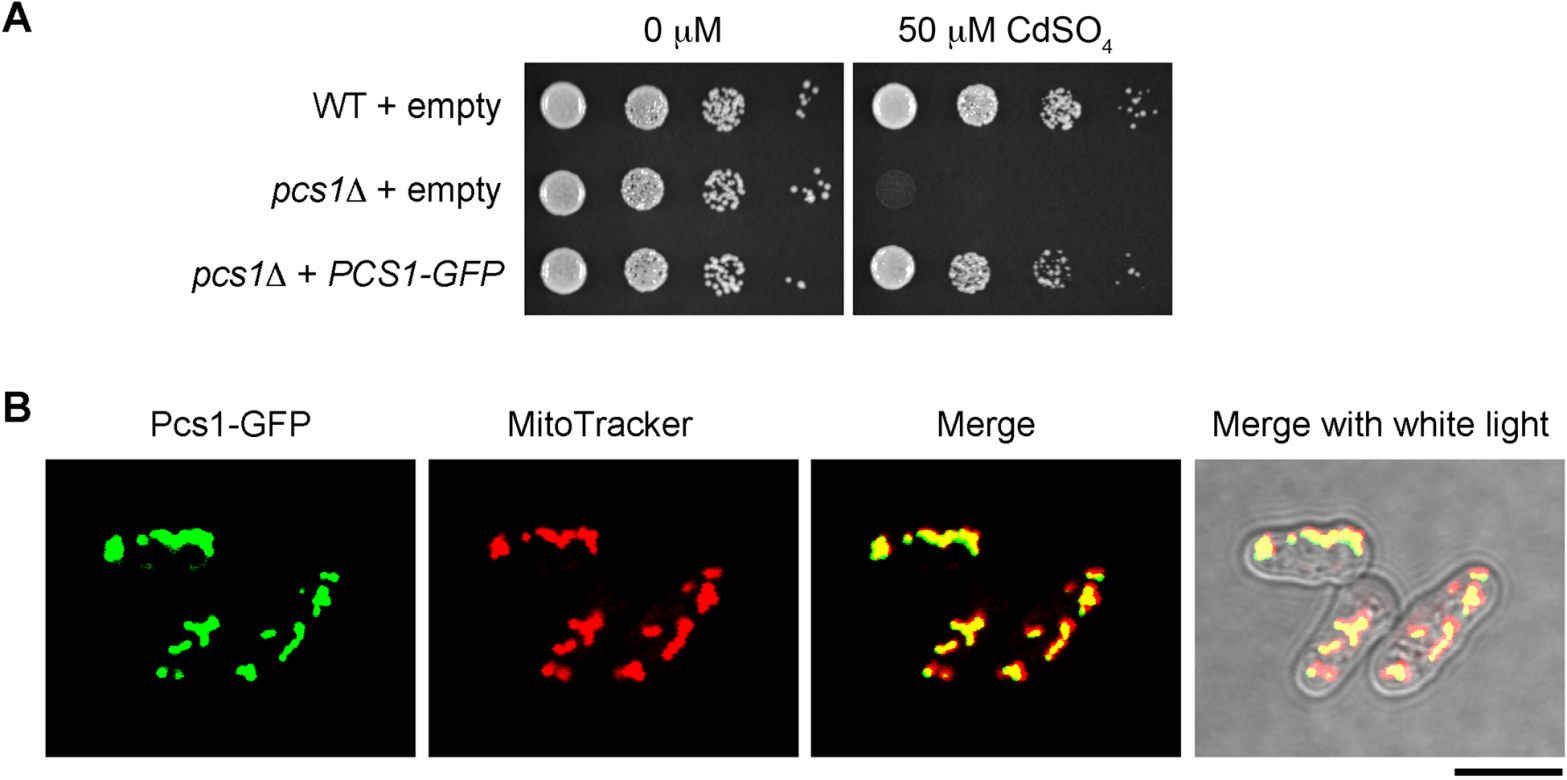
**Pcs1 is targeted to the mitochondria. (A)** 10-fold serial dilutions of *S. pombe* strains AS09, AS12 and AS15 from top to bottom on yeast nitrogen base media with or without CdSO_4_ (4 d at 22°C). The starting wild type strain is the *ura4* mutant, and “empty” indicates the strain carrying the plasmid pREP42. **(B)** Co-localization of Pcs1-GFP in *S. pombe* with MitoTracker red (strain AS15). Scale bar = 10 μm.

As an alternative strategy, the localization of the *S. pombe* PCS protein was explored. The *S. pombe PCS1* homolog was fused to GFP by PCR, the construct cloned, and the plasmid was transformed into the *S. pombe pcs1*Δ strain. The fusion construct complements the cadmium sensitive phenotype (Figure 4A). In the strain expressing this construct, GFP was found in punctate dots or filaments. The pattern of fluorescence co-localized with the mitochondrial dye MitoTracker (Figure 4B), indicating that Pcs1 is targeted to the mitochondria.

### Phytochelatin synthase is widely distributed in the fungi

The genome sequences of fungi were analyzed for the presence or absence of homologs of PCS. Many lineages in the ascomycetes and basidiomycetes did not encode a copy. However, homologs were found in the Pezizomycetes and Taphriniomycotina (Ascomycota) and Pucciniomycotina (Basidiomycota) (Table 1). For the lineages outside of the monophyletic Dikarya, the presence of a PCS was common as it was absent from only two clades examined (the Microsporidia, known for highly reduced genome sizes and gene content, and the one species of Entomophthoromycotina with an available genome sequence). The distribution is consistent with an origin of the gene early in the evolution of the fungi, and then loss on a number of occasions. Major losses in the Basidiomycota would have been in the lineages that gave rise to the Agaricomycotina and Ustilaginomycotina. The presence of homologs in *Wallemia* species, considered either as a sister clade or a basal member of the Agaricomycotina [23,24], could imply two losses. Another major loss was in the Ascomycota lineage that branched after the divergence of the class Pezizomycetes from the other species in the Pezizomycotina phylum. Other losses (e.g. in the Saccharomycotina or the obligate parasites) are consistent with gene fates in these lineages.

**Table 1:** **Distribution of PCS homologs in the fungi**

**Lineage**	**Sub-lineage**	**Genome sequences examined**	**Present/absent?**	**Representative(s) in phylogeny (Figure** 5 **)**
Chytridiomycota (P)		2	Present	*Catenaria*, *Spizellomyces*
Neocallimastigo mycota (P)		1	Present	*Piromyces*
Blastocladiomycota (P)		2	Present	*Allomyces*, *Batrachochytrium*
Microsporidia (P)		5	Absent	
Glomeromycota (P)		1	Present	*Rhizophagus*
Mucoromycotina (SP)	Mucorales (O)	8	Present	*Mucor*, *Rhizopus*, *Phycomyces, Lichtheimia*
	Mortierellales (O)	1	Present	*Mortierella*
Entomophthoro mycotina (SP)		1	Absent	
Zoopagomycotina (SP)		0	Unknown	
Kickxellomycotina (SP)		1	Present	*Coemansia*
Ascomycota (P)	Taphrinomycotina (SP)	7	Present	*Schizosaccharomyces*, *Saitoella*
	Saccharomycotina (SP)	35	Absent	
	Pezizomycotina (SP)	180	Present only in class Pezizomycetes; absent in all other species	*Ascobolus*, *Tuber*, *Wilcoxina*, *Pyronema*
Basidiomycota (P)	Agaricomycotina (SP)	103	Absent	
	Pucciniomycotina (SP)	16	Present	*Sporobolomyces*, *Rhodotorula*, *Puccinia*
	Ustilaginomycotina (SP)	8	Absent	
	Wallemiomycetes	2	Present	*Wallemia*
Unclassified	Cryptomycota	1	Present	*Rozella*
	Monoblepharido mycetes	1	Present	*Gonapodya*

The classification system is based on [56]. P = phylum; SP = subphylum; O = order.

The amino acid sequences of candidate PCS proteins from fungi were downloaded and aligned prior to making phylogenetic trees. After examining alignments of the full amino acid sequences, the variable N and C terminal ends were removed due to their poor alignments. However, both ends should be important for function. The N-terminus is likely involved in subcellular targeting in some species and/or could have a role in protein activity. The C terminus in many homologs is cysteine rich, but the positions of the residues do not align. For example, *S. pombe* has within its C terminus CCX_5_CCX_3_CC; *Sporobolomyces* has CCX_8_CXC; one *Phycomyces blakesleeanus* homolog has CX_2_CX_3_C; and *Catenaria anguillulae* has CCCX_11_CXCC (X indicates any amino acid residue).

**Figure 5:**
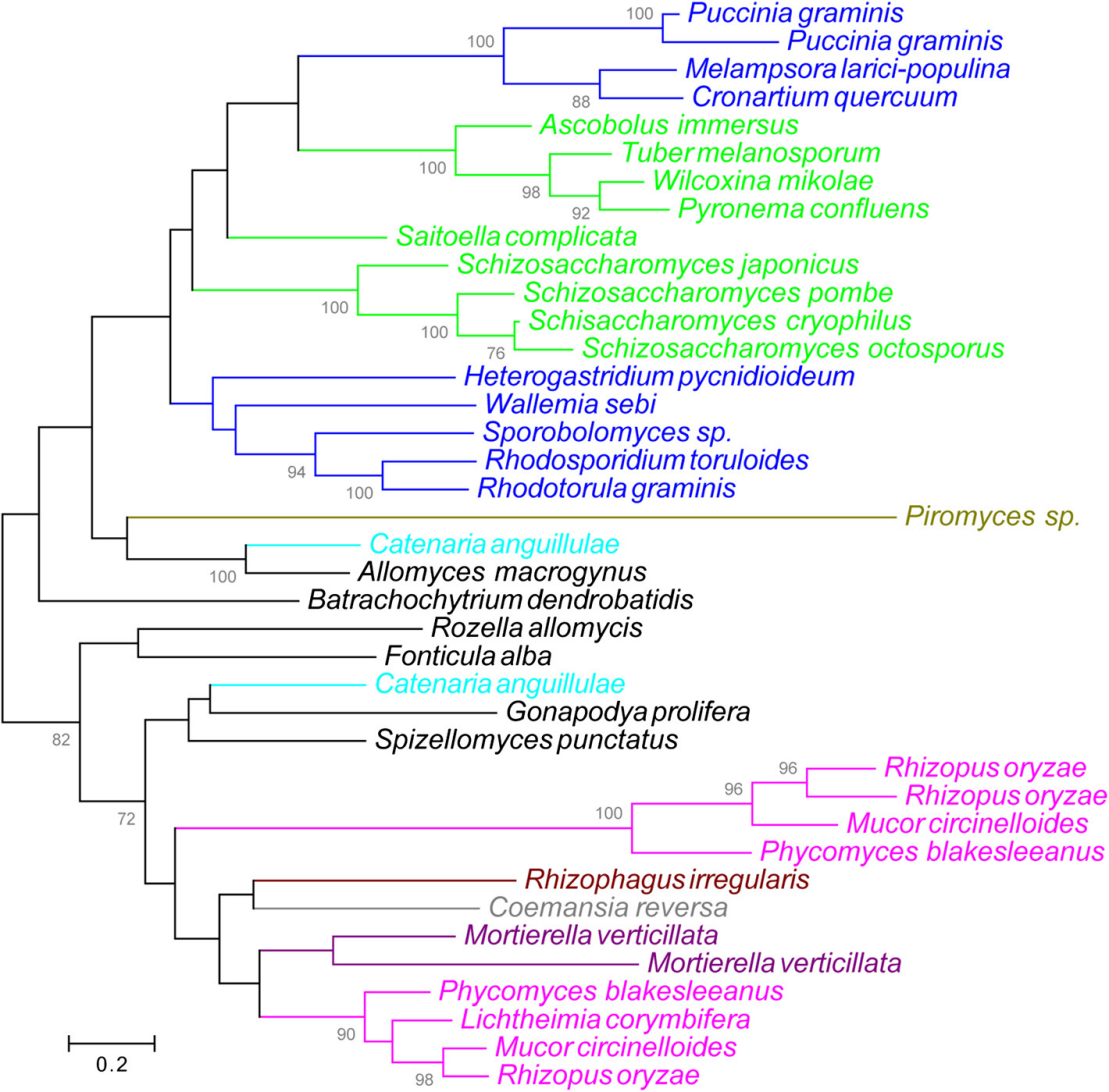
**Phylogeny of PCS proteins from the fungi.** The predicted amino acids were aligned, and a common region spanning 243 residues was compared by maximum likelihood analysis. Numbers above the nodes indicate percentage bootstrap support from 100 reiterations if 70% or higher. Species in the same lineages are color-coding where possible, and their classification is provided in Table 1. *Fonticula alba* is a cellular slime mold (non-fungal).

To build additional support for an early origin for the PCS in fungi, phylogenies were constructed from the fungal homologs, and one maximum likelihood analysis is illustrated in Figure 5. The relationships between many of the homologs are poorly supported based on bootstrapping methods, which is to be expected because of the lack of resolution amongst the basal lineages of the fungi based on conserved housekeeping genes [25]. Nevertheless, distinct groups with high bootstrap support are detected, many of which arrange the PCS homologs within groups of species with monophyletic origins. Other cases were more complicated. For example, the Basidiomycota have two groups that are split by homologs from the Ascomycota, and may indicate horizontal transfer of a gene between lineages.

The amino acid alignment also revealed that four candidate PCS proteins in the Mucoromycotina have amino acid substitutions in the cysteine residue within the active triad site of characterized phytochelatin synthases [26-28]. This draws into question whether these proteins function as enzymes or if they play some other role in these species. In the phylogeny, the four PCS-like proteins form a clade with 100% bootstrap support.

While most ascomycete and basidiomycete species have no PCS, other fungal species show an expansion in copy number. For example, two homologs are in *Puccinia graminis* (Basidiomycota) as a tandem gene duplication in the genome. Most of the Mucoromycotina species have more than one copy, which is similar to a general expansion in gene number through whole genome or segmental genome duplications [29,30]. The duplication dates prior to the separation of these species. Curiously *Catenaria anguillulae* has two putative PCS proteins that are positioned in distinct parts of the phylogeny, which would suggest an interspecies exchange event occurred during its history. The evidence for expansion in copy number would support a hypothesis that the PCS candidates provide a selective advantage to these species, rather than being a vestigial property found in the earlier lineages of the fungi.

## Discussion

Balancing metal homeostasis is crucial for cells to survive and function properly. Organisms must adapt to the environmental availability of such elements, either to take up as much as needed under depleted conditions or to avoid the toxic effects of higher concentrations. Molecules that chelate or sequester metal ions play key roles in these processes.

The emphasis on the function of phytochelatin synthases is their role in protection against the toxic effects of heavy metals, especially cadmium [15,31,32]. However, the distribution of the genes encoding putative phytochelatin synthases in organisms, and the locations of these organisms in nature – here the discussion being on members of the fungal kingdom, but similar points have been raised for plants and other organisms (e.g. [6,33]) – is not consistent with this being their primary role. Heavy metal concentrations can certainly be high in parts of the environment; for example, an estimated 140 million people drink water contaminated by detrimental concentrations of arsenic [34]. However, for many of the fungal species that encode potential phytochelatin synthases these have not been isolated from environments that have such levels of metals. For instance, the *Sporobolomyces* sp. strain used in this study was isolated from the leaf surface of a willow tree [35], although Pucciniomycotina yeasts have been isolated from natural and contaminated metal sites with high levels of cadmium or other metals (e.g. [36-38]). One hypothesis is that the phytochelatins may play a primary role in the homeostasis of essential metals, such as copper or zinc that, while essential, may reach toxic concentrations.

One example of an essential metal that has an interaction with PCS is copper. For instance, in *S. pombe* most focus has been on the cadmium responses mediated by PCS, but there are at least two copper-dependent effects when the gene is mutated. One is on growth rate and the second is on colony color. The source of this pigmentation is unknown, but based on studies in *S. cerevisiae* it is likely due to precipitation of copper sulfite in the cell wall, as occurs in *S. cerevisiae* and where the pigmentation is influenced by cysteine and glutathione levels that are the precursors of phytochelatins [39,40].

A disadvantage of using phytochelatins to protect against metals compared to a mechanism of exclusion or secretion is that the metal remains within the cell. A cell would therefore have limited ways to avoid accumulating a potentially hazardous element within itself. An alternative hypothesis has been proposed in metal hyperaccumulating plants, that toxic metals may serve to protect those species against herbivory (for reviews, see [41,42]). A similar hypothesis is worth exploring for fungi, since certain species produce macroscopic sexual fruiting structures that are eaten and yeasts can be consumed by insects and nematodes. That said, counter to this hypothesis is that the truffle fungus *Tuber melanosporum* and other truffle genomes encode a phytochelatin synthase [8], yet this has not prevented them from become one of the world’s most prized and expensive foods.

Phytochelatin synthase localization in the mitochondria has been proposed based on the presence of a putative targeting sequence at the N-terminal end of the homolog from helminth parasite *Schistosoma mansoni* [17,18]. Alternative splicing produces two isoforms that are predicted to be mitochondrial and cytoplasmic [17]. The full-length (putative mitochondrial) isoform is inactive when expressed in *Escherichia coli*, whereas removing the N-terminus yields an active enzyme [18]. The localization of PCS proteins in eukaryotic cells has not been explored extensively, so we examined the localization of Pcs1-GFP fusion proteins. In *S. pombe*, the Pcs1 protein localized to the mitochondria based on the co-localization of the Pcs1-GFP fluorescence with a standard mitochondrial dye. Analysis of other putative PCS proteins using prediction software also identifies candidate N-terminal mitochondrial targeting sequences in other homologs, from plants like *A. thaliana* to animals like *Caenorhabditis elegans*. However, computation predictions have limitations in their accuracy. As one example, the two PCS proteins in *A. thaliana* are distributed in the cytoplasm with no evidence of targeting to the mitochondria [43]. Alignment of the protein sequences from five Pcs1 homologs and previous characterization of the catalytic regions indicates that the predicted cleavage site to remove the putative mitochondrial targeting region lies within the conserved regions, whereas the fungal species and *S. mansoni* have an extension in the N-terminus that accommodates the predicted mitochondrial targeting sequences (Additional file 1: Figure S1). The presence of PCS in the mitochondria in *S. pombe*, and potentially other organisms, is interesting from the perspective of where in the cell phytochelatins would be synthesized, where they would be required to chelate metals, and to have an idea about the intracellular transport of the phytochelatin-metal complex into vacuoles [44].

Analysis of the distribution of candidate phytochelatin synthase in fungal genomes reveals a presence throughout many lineages. This parallels recent observations suggesting that putative phytochelatin synthases are present in several of the early land plants and in many animal lineages, and may therefore be an ancestral property in each of these eukaryote groups [45-48]. The most obvious losses in the fungi appear in the two most species-rich lineages, the ascomycetes and basidiomycetes. It is unclear why so many of these species have lost this gene, and what would have taken its place. Another protein class that is important for chelation of metals is the metallothioneins. One hypothesis is that during evolution other mechanisms have taken over the control of balancing metal concentrations in those fungi without the PCS homologs.

## Conclusions

The experiments reported here and the analysis of a phytochelatin synthase gene from a basidiomycete species extends the distribution of these enzymes into a second major lineage of the fungi, and shows that they have a conserved function in protection against toxic concentrations of metals. The analysis of fungal genomes, especially those of species other than members of the ascomycetes and basidiomycetes and that are often considered “basal” in the kingdom, demonstrates the presence of homologs widely. The functions of these homologs, in addition to protection against heavy metals like cadmium, are likely in other aspects of metal homeostasis, and remain to be explored in these organisms, as does the potential application of PCS of species with the gene for instance in bioremediation of contaminated sites.

## Methods

### Strains

*Sporobolomyces* sp. strain IAM 13481 was used as the wild type. The *ura5* auxotroph strain AIS2 was isolated previously [49], and was the recipient for gene replacement experiments. *Saccharomyces cerevisiae* strain BY4743 and the *yap1::KanMX* gene replacement strain in this genetic background were used as host strains for expression of the *Sporobolomyces PCS1* homolog. The *Schizosaccharomyces pombe* strain MM72-4A is an *ura4* mutant, derived from wild type L972. More details about the strains are provided in Table 2.

**Table 2:** **Fungal strains used in this study**

**Name**	**Genotype**	**Parent/origin**	**Reference**
*Sporobolomyces* sp.			
IAM 13481	Wild type		[35]
AIS2	*ura5*	IAM 13481	[49]
AS1	*pcs1::URA5 ura5*	AIS2	This study
AS2	*pcs1::ura5 ura5*	AS1	This study
AS3	*pcs1::ura5 ura5 + PCS1-URA5*	AS2	This study
*Saccharomyces cerevisiae*			
BY4743	*MAT* **a**/α *his3*Δ*1*/*his3*Δ*1 leu2*Δ*0*/*leu2*Δ*0 LYS2*/*lys2*Δ*0 met15*Δ*0*/*MET15 ura3*Δ*0*/*ura3*Δ*0*		[57]
*yap1*	*yap1::KanMX*	BY4743	Open Biosystems
AS4	+ pYES2	BY4743	This study
AS5	+ pAS1	BY4743	This study
AS6	+ pYES2 *yap1::KanMX*	*yap1*	This study
AS7	+ pAS1 *yap1::KanMX*	*yap1*	This study
*Schizosaccharomyces pombe*			
L972	Wild type		NBRP, Japan
MM72-4A	*ura4-D18*		NBRP, Japan
AS8	*pcs1::KanMX ura4*	MM72-4A	This study
AS9	+ pREP42	MM72-4A	This study
AS10	+ *S. pombe PCS1* (pAS4)	MM72-4A	This study
AS11	+ *Sporobolomyces PCS1* (pAS5)	MM72-4A	This study
AS12	*pcs1::KanMX* + pREP42	AS8	This study
AS13	*pcs1::KanMX + S. pombe PCS1* (pAS4)	AS8	This study
AS14	*pcs1::KanMX* + *Sporobolomyces PCS1* (pAS5)	AS8	This study
AS15	*pcs1::KanMX* + *PCS1-GFP* (pAS6)	AS8	This study

The *S. cerevisiae* genotypes are abbreviated after their first use in strain BY4743.

### Expression of *Sporobolomyces PCS1* in ascomycete yeasts

The cDNA of *PCS1* from *Sporobolomyces* was amplified using primers AS005 and AS006 from cDNA that was reverse transcribed from total RNA using an oligo-dT primer with Superscript III (Invitrogen, Grand Island, NY, USA). Primers in this study were obtained from Integrated DNA Technologies (Coralville, IA, USA), and their sequences are provided in Table 3. The amplicon was digested with KpnI and XbaI restriction enzymes, and ligated into the same sites in plasmid pYES2 that enables expression in *Saccharomyces cerevisiae* under the control of a galactose-inducible promoter (Invitrogen). A plasmid, pAS1, was identified with no errors by DNA sequencing. The cDNA was amplified from this plasmid with primers AS016 and AS017 and cloned into pCR2.1 (Invitrogen). An insert without errors was identified by sequencing (plasmid pAS2), it was digested with NdeI, and the *PCS1* fragment ligated into the NdeI site of plasmid pREP42 to form plasmid pAS3. The *S. pombe PCS1* open reading frame was amplified from genomic DNA with primers AS018 and AS019 and cloned into pCR2.1. An error-free insert was identified (plasmid pAS4) and excised with BamHI and NdeI, and ligated into the BamHI-NdeI site of pREP42 to form plasmid pAS5. The pREP42 plasmid allows expression in *S. pombe* from a mid-strength *nmt* promoter [16]. The plasmids were transformed into *S. cerevisiae* or *S. pombe* uracil auxotrophic strains using the lithium acetate/PEG method, and selected on yeast nitrogen base (YNB) medium.

**Table 3:** **Primers used in this study**

**Name**	**Sequence (5′-3′)**	**Purpose**
AS008	gtaacgccagggttttcccagtcacgacgCTGATTTCTGCGAAGAGG	Disruption of *Sporobolomyces PCS1*
AS002	GGTCTTTCCAGGGAGAGAGGGTTCATTCTGAGTGAG	
AS003	CAAGTAGAACGAAGGGTTCGCATTCTCAACCATCTC	
AS009	gcggataacaatttcacacaggaaacagcCAAGAGTTGTTCGGTGCG	
AS005	GG*GGTACC*ATGACGCTTGCCACCAAAGC	Cloning *Sporobolomyces PCS1* cDNA
AS006	GC*TCTAGA*CTAGTGACAACCCTTCTGC	
ALID0562	TCTCTCCCTGGAAAGACC	Amplification of *URA5* selectable marker
ALID0564	AACCCTTCGTTCTACTTG	
AS012	TCGGTTATCGGTTAAGGC	Gene replacement in *S. pombe*
AS013	GTCGACCTGCAGCGTACGTCCCTCTTGCAATGCTCG	
AS014	CGAGCTCGAATTCATCGATTCCCAAAGGCGTTCTAG	
AS015	TAAACGAAAGCACGAGCG	
KanMX F	CGTACGCTGCAGGTCGAC	
KanMX R	ATCGATGAATTCGAGCTCG	
AS016	GCC*CATATG*ACGCTTGCCACCAAAGC	*Sporobolomyces PCS1* for expression in *S. pombe*
AS017	GCC*CATATG*CTAGTGACAACCCTTCTG	
AS018	CGG*CATATG*AACATTGTTAAACGAG	Amplifcation of *S. pombe PCS1*
AS019	ATT*GGATCC*TCACGTATTTTTACAGCAG	
ALID2091	CAAGCTGCTGTAAAAATACGATGGTGAGCAAGGGCGAG	GFP localization of *S. pombe* Pcs1
ALID2092	CTCGCCCTTGCTCACCATCGTATTTTTACAGCAGCTTG	
AISV066	CCG*GGATCC*TTACTTGTACAGCTCGTC	
AS004	CAAGAGTTGTTCGGTGCG	Wild type *Sporobolomyces PCS1* for complementation
AS020	GGTCTTTCCAGGGAGAGACTGATTTCTGCGAAG	
ALID1432	GAGTACATGGTCTACATG	*GPD1* for northern blots
ALID1433	AAGGCAATGTGGCAGAGG	

Letters in italics are restriction enzyme cut sites introduced into the DNA sequences. Lower case letters indicate regions that were included for possible use for *in vivo* recombination into *S. cerevisiae* plasmids.

### Targeted gene replacement of *PCS1* homologs in *Sporobolomyces* and *Schizosaccharomyces*

The *PCS1* gene of *Sporobolomyces* was replaced with a *URA5* selectable marker by homologous recombination. A construct with 1,334 and 1,232 bp on either side of the wild type *URA5* gene was created by forming a three-part DNA fragment with overlap PCR. The flanks of the *PCS1* gene were amplified with primers AS001-AS002 and AS003-AS004. The *URA5* gene was amplified with primers ALID0562-ALID0564. The three fragments were mixed, and an overlap fragment amplified with primers AS001-AS004. The construct was transformed into cells of the AIS2 uracil auxotrophic strain (a *ura5* mutant) plated on yeast nitrogen base agar + 1 M sorbitol, by biolistic delivery of the DNA coated onto gold beads, as described previously [49]. A strain with the targeted gene replacement was identified by PCR analysis. To complement the *pcs1*Δ mutation, first a spontaneous mutation in the *URA5* gene of the *pcs1::URA5 ura5* strain was isolated by plating on 5-fluoroorotic acid medium. The full-length *PCS1* gene that includes the promoter and terminator regions was amplified from wild type DNA using primers AS004-AS020, and *URA5* amplified with primers ALID0562-ALID0564. A two-fragment overlap PCR was performed using primers AS004-ALID0564. This construct was transformed into the *pcs1 ura5* mutant strain by biolistic delivery of the DNA and transformants were selected on yeast nitrogen base medium.

The *pcs1* gene of *S. pombe* was also replaced by homologous recombination. A construct with 360 and 339 bp on either side of the *KanMX* cassette was created by overlap PCR. The 5′ flank was amplified with primers AS012 and AS013, the 3′ flank with AS014 and AS015, and the *KanMX* selectable marker with primers KanMX F and KanMX R. Equimolar amounts were mixed, and the overlap PCR performed with primers AS012-AS015. The product was transformed into strain MM72-4A using the lithium acetate/PEG method. Cells were recovered overnight in liquid medium (5 g/L yeast extract, 10 g/L peptone, 3% glucose), and plated as aliquots onto the same medium that included agar and containing 50 or 100 μg/L G-418. A gene replacement strain was identified by PCR analysis.

### Nucleic acid manipulations

Genomic DNA was isolated using a CTAB buffer extraction protocol [50]. RNA was isolated using TRI Reagent (Sigma-Aldrich, St. Louis, MO, USA). Nucleic acids were blotted to Zeta-Probe membrane (Bio-Rad, Hercules, CA, USA), and probed with ^32^P-dCTP labeled DNA fragments using standard methods. As a loading and transfer control for northern blots, a fragment of the constitutively-expressed *GPD1* gene, encoding glyceraldehyde-3-phosphate dehydrogenase, was amplified with primers ALID1432-ALID1433 for use as a probe.

### Subcellular localization using GFP fusions

The *PCS1* gene from *S. pombe* was fused in frame at the C-terminal to the gene encoding green fluorescent protein. The *PCS1* gene was amplified with primers AS018-ALID2092. GFP was amplified with primers AISV066-ALID2091. An overlap PCR product was amplified with primers AS018-AISV066, and this product cloned into plasmid pCR2.1. The construct was excised from the pCR2.1 plasmid with BamHI-NdeI and cloned into the BamHI-NdeI site of plasmid pREP42 to make plasmid pAS6. The plasmid was transformed into the *S. pombe pcs1*Δ strain AS8 with the lithium acetate/PEG method, and transformants were selected on YNB.

The yeast strains were cultured in Edinburgh Minimal Medium (MP Biomedicals, Solon, OH, USA) and stained with MitoTracker red (Invitrogen), at 3 nM, with the staining of the cells resuspended in water for 20 min. Cells were washed and then re-suspended in phosphate buffered saline. Fluorescence was examined using an Olympus Fluoview FV300 confocal microscope.

### Phylogenetic, genome sequence, and other computational analyses

Putative phytochelatin synthases were sought by BLASTp queries against the MycoCosm (US Department of Energy), NCBI, and Broad Institute databases [51,52]. The last queries were made on 1 November 2014. Protein sequences were aligned with ClustalW and the alignment inspected by eye. Protein predictions were examined for unusual gaps or sequence divergences that may reflect annotation errors. Revised annotations were generated in six cases: for *Pyronema confluens*, *Rhizophagus irregularis*, *Tuber melanosporum*, *Rhizopus oryzae*, *Batrachochytrium dendrobatidis*, and an alternative model in the MycoCosm database selected for *Phycomyces blakesleeanus*. After constructing a new alignment, the N and C terminal variable ends were removed, yielding an alignment of 243 amino acids. The alignment is provided in the Additional file 2. Evolutionary relationships between PCS proteins were explored using MEGA6 [53]. The best-fit model for maximum likelihood was selected, and a phylogeny was constructed using maximum likelihood (LG + G; using all amino acid sites). 100 bootstraps were used to infer support for the nodes within the tree.

For predicting subcellular localization of Pcs1 homologs, the MitoProt and Psort II programs were used [54,55].

## Availability of supporting data

The *Sporobolomyces* sp. *PCS1* genomic and cDNA sequence information, defined by dideoxy sequencing during this study, was deposited to GenBank as accession KJ000020.

## Additional files

Additional file 1: Figure S1. Comparison of the structures of four characterized phytochelatin synthases with the *Sporobolomyces* sp. predicted protein. The proteins feature a conserved core that contains the catalytic site, illustrated as a black box with the % amino acid identities and % similarities in parentheses (BLOSUM62 matrix) compared to *Sporobolomyces* Pcs1 listed. The conserved region is flanked on the N and C terminal ends by non-conserved regions of variable length.

Additional file 2: **Alignment of a fragment of the predicted amino acid sequences of putative phytochelatin synthase homologs in fungi.**
