# Supplementary figures and images for: Phytochelatin synthase is required for tolerating metal toxicity in a basidiomycete yeast and is a conserved factor involved in metal homeostasis in fungi
Source: Fungal Biol Biotechnol. 2015 Mar 28;2:3. doi: 10.1186/s40694-015-0013-3 (PMC4410428; doi:10.1186/s40694-015-0013-3)

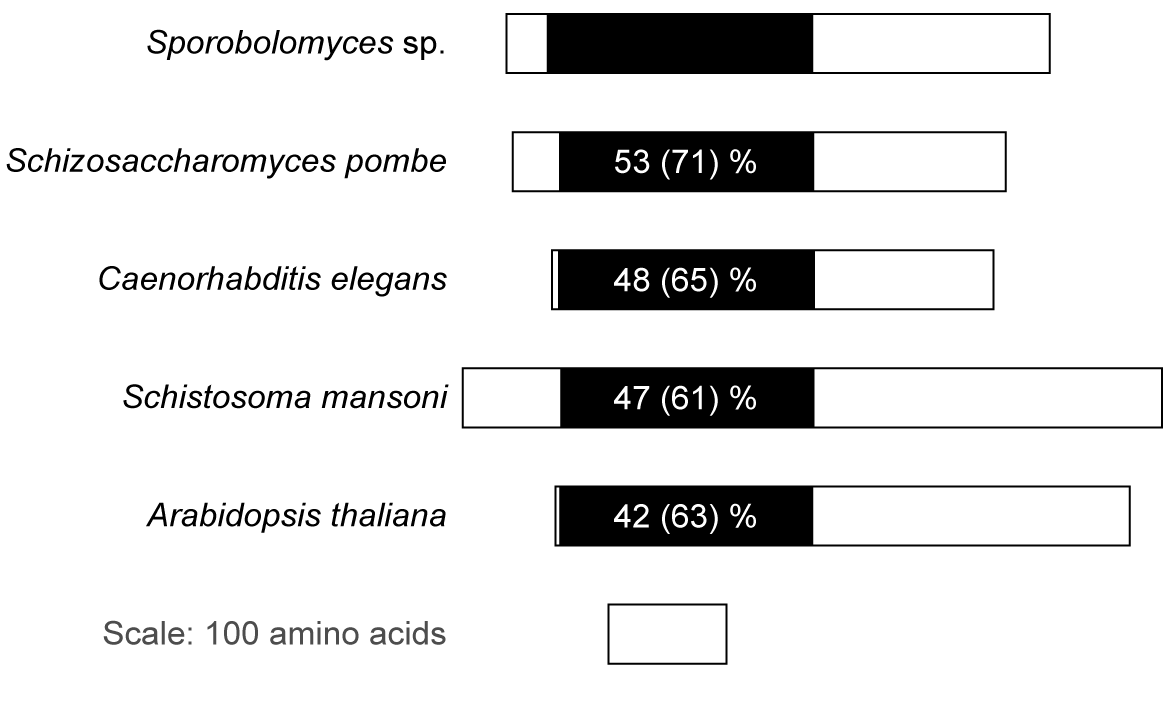

Supplement: Supplementary file 1 — Comparison of the structures of four characterized phytochelatin synthases with the Sporobolomyces sp. predicted protein. The proteins feature a conserved core that contains the catalytic site, illustrated as a black box with the % amino acid identities and % similarities in parentheses (BLOSUM62 matrix) compared to Sporobolomyces Pcs1 listed. The conserved region is flanked on the N and C terminal ends by non-conserved regions of variable length. [file 40694_2015_13_MOESM1_ESM.tiff]
